# The Use of Waste from the Production of Rapeseed Oil for Obtaining of New Polyurethane Composites

**DOI:** 10.3390/polym11091431

**Published:** 2019-08-31

**Authors:** Joanna Paciorek-Sadowska, Marcin Borowicz, Marek Isbrandt, Bogusław Czupryński, Łukasz Apiecionek

**Affiliations:** 1 Department of Chemistry and Technology of Polyurethanes, Technical Institute, Faculty of Mathematics, Physics and Technical Science, Kazimierz Wielki University, J. K. Chodkiewicza Street 30, 85-064 Bydgoszcz, Poland; 2 Department of Computer Science, Technical Institute, Faculty of Mathematics, Physics and Technical Science, Kazimierz Wielki University, J. K. Chodkiewicza Street 30, 85-064 Bydgoszcz, Poland

**Keywords:** rigid polyurethane-polyisocyanurate foams, biofiller, polyurethane properties, rapeseed cake

## Abstract

This article presents the results of research on obtaining new polyurethane materials modified by a by-product from vegetable oils industry—rapeseed cake. The chemical composition of rapeseed cake was examined. Rigid polyurethane-polyisocyanurate (RPU/PIR) foams containing a milled rapeseed cake in their composition were obtained as part of the conducted research. Biofiller was added in amount of 30 wt.% up to 60 wt.%. Effects of rapeseed cake on the foaming process, cell structure and selected properties of foams, such as apparent density, compressive strength, brittleness, flammability, absorbability, water absorption, thermal resistance and thermal conductivity are described. The foaming process of RPU/PIR foams modified by rapeseed cake was characterized by a lower reactivity, lower foaming temperature and decrease in dielectric polarization. This resulted in a slowed formation of the polyurethane matrix. Apparent density of RPU/PIR foams with biofiller was higher than in unmodified foam. Addition of rapeseed cake did not have a significant influence on the thermal conductivity of obtained materials. However, we observed a tendency for opening the cells of modified foams and obtaining a smaller cross-sectional area of cells. This led to an increase of absorbability and water absorption of obtained materials. However, an advantageous effect of using rapeseed cake in polyurethane formulations was noted. Modified RPU/PIR foams had higher compressive strength, lower brittleness and lower flammability than reference foam.

## 1. Introduction

Oil crops, after cereals, are the second most important source of energy in consumed foods by humans. They are used also as a feed for farm animals. In addition, they are also a valuable source of raw material for many industrial products [1]. Soy has the largest share in the global production of oilseeds (about 57%). The next places are occupied by rapeseed (20%), cotton (11%) and sunflower (8%) [2]. 

A particularly important oil plant in the temperate climate zone is rapeseed (*Brassica napus*), whose production shows an increasing tendency both in Europe and in the world. Rapeseed is grown on an area of over 3 million hectares in Europe, which is over 60% of the total area occupied by oilseeds. This plant is an important source of fat (oil). The oil obtained from rapeseed has very valuable qualities and it is used in many industries. Rapeseed oil is particularly appreciated in the food and cosmetic industry due to its high content of vitamins, unsaturated fatty acids, its excellent taste and health attributes. Rapeseed oil is also used as an additive for diesel fuel or as an independent fuel [3,4]. 

The by-product of the production of vegetable oils are pomace (so-called oil cake). Until now, their most important application was use in production of animal feed [5]. However, this by-product obtained in many technologies contains anti-nutritive substances (e.g., glucosinolates, synapin, protease inhibitors, phytates and erucic acid), which negatively affect feed intake, animal growth and their metabolism [6,7,8]. Therefore, the use of rapeseed cake in animal nutrition must be very limited. Problem of oil cake management begins to increase in connection with the implementation of biofuel technologies. Rapeseed cake can be also a potential organic material used as a biofuel for heating purposes. However, rapeseed cakes should be assessed as a moderately good fuel material due to the high content of protein and amino acids. These compounds generate during combustion a high emission of nitrogen and sulfur oxides, which in turn is harmful to the environment. Therefore, the correctness of combustion of these substances must take into account the possibility of tar and soot formation during pyrolysis [9].

Increasing demand for vegetable oils in the last decade results not only from the improvement of the nutrition of societies, but also is the consequence of the production development of oleochemical raw materials [10,11,12,13,14]. Proper early development of innovative methods for the disposal of oil cakes will prevent possible problems with their overproduction. Hence the concept of our work, based on the use of rapeseed cake as a biofiller for production of polyurethane composites.

Polyurethanes are a group of polymeric materials with the most versatile properties, applicable in every area of life and the economy [15,16,17,18]. These polymers are among the most important modern plastics with a total consumption of more than 12 million tons per year. Polyurethanes displace traditional materials such as rubber, metals and ceramics, which is a result of their favorable mechanical properties and high chemical resistance [19,20].

Rigid polyurethane foams (RPUFs) are characterized by the best thermal insulation properties among the currently available polymeric materials. The value of thermal conductivity coefficient of these materials ranges from 0.018 to 0.025 W/(m·K) and is significantly lower than values of this parameter for other known thermal insulation materials like expanded polystyrene and mineral wool. Considering two basic criteria for the selection of filler, i.e., availability and price, it is reasonable to use rapeseed cake, because the overproduction of it becomes a problem. In addition, the use of a by-product obtained from renewable raw materials is considered one of the possible methods for balancing economic and ecological imperatives [21,22].

The modern polyurethane industry is focused on pro-ecological activities. Therefore, there is currently an intensive development of technologies based on environmentally friendly raw materials. These raw materials include, e.g., biofillers derived from the processing of natural resources. They constitute a wide group of solid additives modifying the properties of the polyurethane materials. They can be divided into powder and fibrous fillers. The purpose of biofiller addition into polyurethane composite is usually to improve or maintain its properties while reducing the price of the finished product. One of the main sources of obtaining biofillers is the wood industry. Many products or waste from wood processing are perfectly suited to the modification of polyurethane materials. They include, among others, lignin and cellulose. Tavares et al. and Pan et al. used Kraft lignin as an active biofiller for production of rigid polyurethane foams [23,24]. Benhamou et al. and Park et al. used nanocellulose in the form of nanocrystals and nanofibers to obtain PUR elastomer composites. In both cases, the authors observed a positive effect of biofiller on mechanical properties and thermal resistance [25,26]. Gama et al. obtained viscoelastic polyurethane foams based on ground coffee. Biocomposites were characterized by high modulus of elasticity, good thermal conductivity and thermal stability. In addition, authors showed that obtained bioplastics exhibit sound insulation ability [27]. Sanches et al. used cotton fibers as a biofiller to obtain aqueous polyurethane dispersions. The material was characterized by higher mechanical strength in a dry environment. However, the properties of this composite deteriorated rapidly after exposure to moisture [28]. Paciorek-Sadowska et al. used the waste oak bark (*Quercus robur L*) as a biofiller for the synthesis of rigid polyurethane-polyisocyanurate foams. The authors observed an increase in thermal resistance and reduced flammability of modified materials [29]. Bakare et al. used sisal fibers with biopolyol based on natural rubber seed oil to produce polyurethane elastomers. Deionized and dried sisal fibers were added into material in an amount of 0 to 30 wt.%. The addition of biofiller increased tensile strength and modulus of elasticity [30]. In turn, Lee and Wang obtained elastomeric biocomposites modified by bamboo fiber. They used biopolyols based on polylactide and poly(butylene succinate) and ecological L-lysine diisocyanate as a reaction system. Obtained biocomposites had high tensile strength and high water resistance. The authors also showed that the use of such reaction system promoted easier biodegradation of the elastomer [31]. Ekici et al. used fibers from tea tree leaf to produce foam with sound insulation properties. Research showed that the biofiller improved sound absorption in the 1.6–6.3 kHz frequency range. It also reduced the cost of the finished product because it was cheap waste [32].

Development directions of new polyurethane materials are increasingly pointing to the use of raw materials from renewable sources. This is due to depletion of fossil resources as well as easier recycling of post-consumer products through biodegradation processes.

The present work meets these expectations and focusses the research on the physicochemical properties of polyurethane biocomposites, which are important from the application point of view. However, it requires the production of PU foams according to newly developed formulations and checking their compliance for various applications.

## 2. Materials and Methods 

### 2.1. Raw Materials

A polyether polyol Rokopol RF-551—product of sorbitol oxyalkylation of hydroxyl number HN = 420 mgKOH/g (produced by ZCh PCC Rokita SA, Brzeg Dolny, Poland)—and technical polyisocyanate Purocyn B (supplied by Purinova Ltd., Bydgoszcz, Poland) were used to obtain RPU/PIR foams. The main component of isocyanate raw material was 4,4′-diphenylmethane diisocyanate. Density of polyisocyanate at 25 °C was 1.23 g/cm^3^, viscosity was 200 mPa·s and free NCO groups content was 31.0 wt.%. Polyether polyol and polyisocyanate met the requirements of ASTM D2849-69 and ASTM D1638-70.

**Figure 1:**
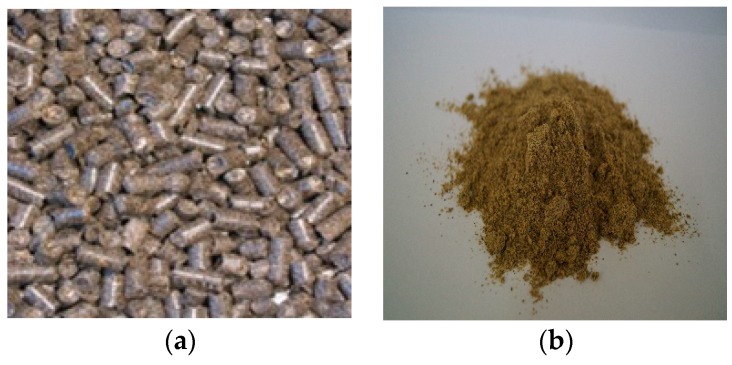
Rapeseed cake: (**a**) pellet, (**b**) milled pellet.

Catalytic system in the process of obtaining RPU/PIR foams was anhydrous potassium acetate (produced by Chempur, Piekary Śląskie, Poland) used in 33% solution in diethylene glycol (produced by Chempur, Piekary Śląskie, Poland) and DABCO (1,4-diazabicyclo (2.2.2) octane, produced by Alfa Aesar, Haverhill, MA, USA) used also in 33% solution in diethylene glycol. Cell structure stabilizer of obtained foams was poly(siloxanyloxyalkylene) surfactant Tegostab 8460 (produced by Evonik, Essen, Germany). Blowing agent was carbon dioxide obtained as a result of the reaction of excess isocyanate raw material with water. Flame retardant was Antiblaze TMCP—tri[2-chloro-1-methylethyl] phosphate (produced by Albemarle, Charlotte, NC, USA). Biofiller used in this research was rapeseed cake (produced by stuja.pl, Bydgoszcz, Poland). This press cake was obtained in the form of a pellet during process of obtaining rapeseed oil using the cold pressing method. Dry and deoiled pellet was ground in a laboratory mill. A powder with a grain diameter below 0.5 mm was obtained. The physical form of rapeseed cake is presented in Figure 1. Selected chemical composition of the ground bio-filler is shown in Table 1.

Oil press cake is a by-product (or waste product) obtained during the pressing of rapeseeds in production of unrefined oil. It is characterized by a high content of fatty acids, natural antioxidants (vitamins, e.g., E, B1, B2, B6, PP, folic acid, biotin and choline), protein (e.g., lysine, methionine, cystine, threonine and tryptophan), minerals (e.g., phosphorus) and crude fibers [33,34]. High fat content and presence of fiber in the cake are due to the degree of embossing rapeseeds, and the content of protein and phosphorus depends on the genetic features of the plant [35,36].

### 2.2. Preparation of RPU/PIR Foams Modified by Rapeseed Cake

Development of RPU/PIR foams with the addition of rapeseed cake required experimental tests to determine the optimal composition of additives (catalysts, surfactant, flame retardant and blowing agent). Hydroxyl number of polyol was used to calculate the amount of this raw material. Amount of isocyanate raw material was calculated taking into account the ratio of isocyanate groups to hydroxyl groups, which was 3:1 for RPU/PIR foams. The calculated amount of isocyanate was increased by an excess of isocyanate necessary to carry out the reaction with water. CO_2_ was released as a result of this reaction. Obtained gas foamed the reaction mixture. The total ratio of isocyanate groups to hydroxyl groups in the obtained systems was always 3.7:1. The next step was to determine the content of additives, which usually do not have hydroxyl groups, i.e., catalysts, non-reactive flame retardant and surfactant. Their contribution was calculated by referring to weight parts per 100 weight parts of polyol. Calculated formulations of obtained foams are shown in Table 2.

The polymerization of urethane plastics includes two main processes taking place simultaneously: additive polymerization of reactive mixture and formation of products from this mixture. An important factor determining the correct course of polymerization is intensive mixing of ingredients mixture before filling the processing mould. The foam formation process is chemically complex. It may be additionally hindered by technological problems that often accompany the process of obtaining polyurethane foams. Therefore, it is necessary to conduct experimental research in order to obtain products with favorable properties.

Preparation of RPU/PIR foams according to the developed formulations was carried out in polypropylene vessels with a volume of 1 dm^3^. Polyisocyanate raw material was weighed in one, while in the other a mixture of the remaining components (polyol with additives). Isocyanate raw material was added to polyol premix and mixed thoroughly with a mechanical stirrer for 10 s. Reaction mixture was poured into a mould after this time, and a free rise process of foams was observed. The used open mould was made of stainless steel with a thickness of 1.5 mm. Internal dimensions of this mould were 25 cm × 25 cm × 30 cm.

A series of RPU/PIR foams was obtained as a result of synthesis. New polyurethane materials were marked with the symbols M0—foam without filler (rapeseed cake), and M30, M40, M50, M60—foams with an increasing percentage amount of filler from 30 wt.% to 60 wt.% (oil cake was increased by 10 wt.%). The percentage participation of biofiller was calculated relative to the sum of mass of polyol and isocyanate raw material. Synthesis of foams was repeated twice. Obtained materials were thermostated within 4 h at 120 °C in a laboratory dryer with forced circulation, after being removed from the mould.

### 2.3. Methods

RPU/PIR–rapeseed cake composites have been tested for their basic functional properties, i.e., foaming parameters, apparent density, brittleness, compressive strength, aging resistance, water absorption and thermal insulation properties.

The course of foaming process was analyzed using the FOAMAT® apparatus (Format-Messtechnik GmbH, Karlsruhe, Germany). This device enabled analysis of a number of parameters during the foaming process, such as a function of time, temperature of process, pressure, dielectric polarization and height of foam rise. The characteristic foaming times, such as cream time (from the start of mixing components A and B until fine bubbles begin to appear), free rise time (from the start of mixing the components A and B until the foam stops expanding), string gel time (from the start of mixing the components A and B until long strings of tacky material can be pulled away from foam surface when the surface is touched by tongue depressor) and tack free time (from the start of mixing components A and B until the foam surface can be touched by tongue depressor without sticking) were also determined using the electronic stopwatch in accordance with ASTM D7487 13e^1^—Standard Practice for Polyurethane Raw Materials: Polyurethane Foam Cup Test.

The apparent density of foams (ratio of foam weight to its geometrical volume) was determined for cube-shaped samples with a side length of 50 mm in accordance with ISO 845:2006.

Compressive strength was determined by using the universal testing machine Instron 5544 (Instron, Norwood, MA, USA) in accordance with ISO 844:2014. The maximum force inducing a 10% relative strain was determined (decreasing of the foam height in relation to the initial height, according to the direction of foam growth).

Analysis of the differential scanning calorimetry (DSC) of RPU/PIR–rapeseed composites was carried out using DSC 204 F1 apparatus (Netzsch Analysing & Testing, Selb, Germany) in atmosphere of inert gas—nitrogen. Temperature range of measurement was from 20 °C to 400 °C. Heating rate was 5 °C/min.

Thermogravimetric analysis (TGA) of foams was made in the temperature range from 20 to 1000 °C. Analysis was carried out under nitrogen at a flow of 2 cm^3^/min. The sample weight was 8 to 10 mg. The measurement was performed using a TGA device from TA Instruments (New Castle, DE, USA). The sample heating rate was 10 °C/min.

Brittleness of the foams was determined in accordance with ASTM C-421-61, as a percentage mass loss of 12 cubic foam samples with a side length of 25 mm. Tests were conducted in a standard cuboidal box made of oak wood with dimensions of 190 mm × 197 mm × 197 mm, rotating around the axis at a speed of 60 rpm. The filling of the box during the measurement were 24 normalized oak cubes with dimensions of 20 mm × 20 mm × 20 mm. Brittleness (B) of obtained foams was calculated from Equation (1):

(1) $$B=\frac{m_{1}-m_{2}}{m_{1}}\cdot 100\%$$

where: *m*_1_—mass of the sample before test (g), and *m*_2_—mass of the sample after test (g).

Aging resistance of foams was carried out in thermostating process of cubic samples with a side length of 50 mm in 48 h at a temperature of 120 °C. The results of this test included a change of linear dimensions (Δ*l*), change of geometrical volume (Δ*V*) and of mass loss (Δ*m*). Values of these parameters were calculated in accordance with ISO 1923:1981 and PN-EN ISO 4590:2016-11. The formulas for the calculations of Δ*l*, Δ*V*, Δ*m* are shown in Equations (2)–(4):

(2) $$\Delta l=\frac{l-l_{0}}{l_{0}}\cdot 100\%$$

where: *l*_0_—length of the sample before thermostating (according to the direction of foam rise) (mm), and *l*—length of the sample after thermostating (according to the direction of foam rise) (mm);

(3) $$\Delta V=\frac{V-V_{0}}{V_{0}}\cdot 100\%$$

where: *V*_0_—geometrical volume of the sample before thermostating (mm^3^), and *V*—geometrical volume of the sample after thermostating (mm^3^);

(4) $$\Delta m=\frac{m_{0}-m}{m_{0}}\cdot 100\%$$

where: *m*_0_—mass of the sample before thermostating (g), and *m*—mass of the sample after thermostating (g).

Thermal conductivity of RPU/PIR foams was determined by measuring the thermal conductivity coefficient (λ). Foam samples with dimensions of 200 mm × 200 mm × 25 mm were tested. FOX 200 apparatus (TA Instruments, New Castle, DE, USA) was used to carry out these tests. This device allowed to specify values in the range of 20–100 mW/(m·K). Measurements are carried out in series at intervals of 0.5 s. Value of thermal conductivity coefficient was calculated from the Fourier Equation (5):

(5) $$q=-\lambda \cdot \frac{dT}{dx}$$

where: *q*—density of the total thermal flux (W/m^2^) transported on the *x* way, *λ*—thermal conductivity coefficient (W/(m·K)) and *dT/dx*—temperature gradient in *x* direction (K/m).

Content of closed cells was determined in accordance with PN-EN ISO 4590:2016-11 by using the helium pycnometer AccuPyc 1340 with the FoamPyc option (Micrometrics, Norcross, GA, USA). Foam samples with dimensions: 25 mm × 25 mm × 130 mm were used for the tests. The foam samples lacked structural defects. The principle of measurement is based on the Boyle–Mariotte law. An increase in gas volume in a closed vessel causes a proportional reduction in pressure according to this law. If the volume of the chamber increases evenly in the presence of or without the sample, the pressure decrease will be lower in the case of an empty chamber. The method consists in determining the pressure change. Percentage of closed cell content in the sample (K_z_) is determined from Equation (6):

(6) $$K_{z}=\frac{V_{z}}{L\cdot w\cdot t}\cdot 10^{2}=\frac{V_{z}}{V_{c}}\cdot 10^{2}$$

where: *V_z_*—closed volume of the sample read from the calibration graph (mm^3^), *L*—average length of the sample (mm), *w*—average width of the sample (mm), *t*—average thickness of the sample (mm) and *V_c_*—total volume of the sample (mm^3^).

Absorbability (*A*) and water absorption (*WA*) were determined in accordance with ISO 2896:2001, which was measured after immersion in distilled water for 24 h. Values of these parameters were calculated from Equations (7) and (8):

(7) $$A=\frac{m_{A}-m_{D}}{m_{D}}\cdot 100\%$$

where: *m_A_*—mass of the sample after immersion in distilled water (g), and *m_D_*—mass of the dry sample (g).

The method of determining water absorption is based on measuring the sample mass after surface drying (determination of water contained inside the foam):

(8) $$WA=\frac{m_{WA}-m_{D}}{m_{D}}\cdot 100\%$$

where: *m_WA_*—mass of the sample after surface drying (g).

Limited oxygen index (LOI) was measured by using Concept Equipment apparatus (Rustington, UK) in accordance with ISO 4589. The percentage limited concentration of oxygen was determined in the mixture consisting of oxygen and nitrogen, which was sufficient to sustain the burning of the sample. *LOI* was calculated according to the Equation (9):

(9) $$LOI=\frac{\left[O_{2}\right]}{\left[O_{2}\right]\;+\;\left[N_{2}\right]}\cdot 100\%$$

where: [*O*_2_]—volumetric flow of oxygen at the limit concentration (m^3^/h), and [*N*_2_]— volumetric flow of nitrogen at the limit concentration (m^3^/h).

Flammability of RPU/PIR composites was also determined by using Bütler’s combustion test (vertical test) in accordance with ASTM D3014-04. Bütler’s combustion test, which consisted of burning a foam sample with the dimensions of 150 mm × 20 mm × 20 mm in a vertical column (chimney) with dimensions of 300 mm × 57 mm × 54 mm. Combustion residue (CR) was calculated from Equation (10):

(10) $$CR=\;\frac{m_{b}}{m_{a}}\cdot 100\%$$

where: *m_a_*—mass of the sample before the burning test (g), and *m_b_*—mass of the sample after the burning test (g).

Foam structure of foams modified by rapeseed cake was analyzed by scanning electron microscope (SEM) HITACHI SU8010 (Hitachi High-Technologies Co., Tokyo, Japan) with the NORAN Vantage microanalysis system. The studies were performed at the accelerating voltage of 10 kV, with the working distance of 10 mm and magnification of 30×. All SEM micrographs were made in a parallel direction to the direction of foam rise. The statistical analysis of cell sizes, wall thickness, and content of cell per area unit was carried out on the basis of obtained micrographs by using ImageJ software (LOCI, Madison, WI, USA).

## 3. Results and Discussion

### 3.1. Foaming Process

**Figure 2:**
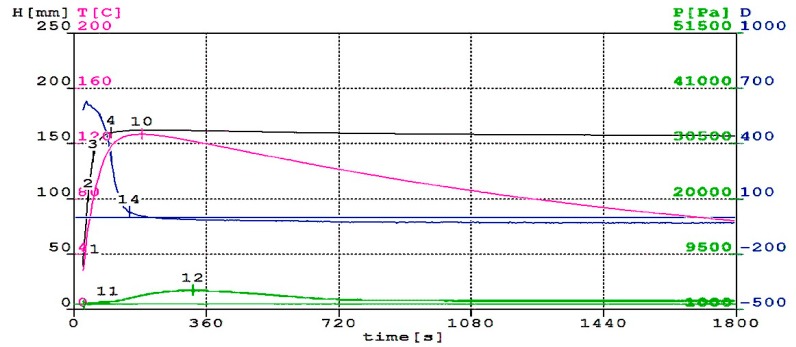
Change in height (H), temperature (T), pressure (p) and dielectric polarization (D) of reaction mixture during foaming process for M0 foam without biofiller.

**Figure 3:**
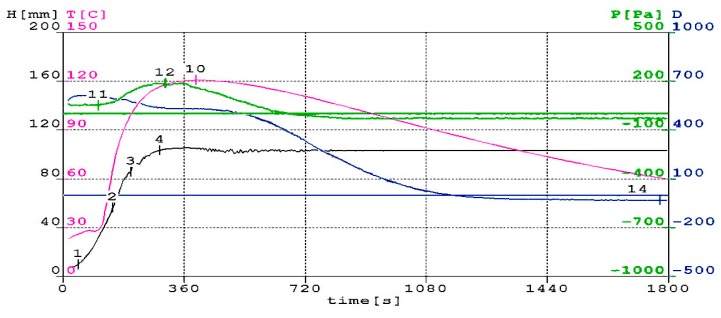
Change in height (H), temperature (T), pressure (p) and dielectric polarization (D) of reaction mixture during foaming process for M30 foam with 30 wt.% of biofiller.

**Figure 4:**
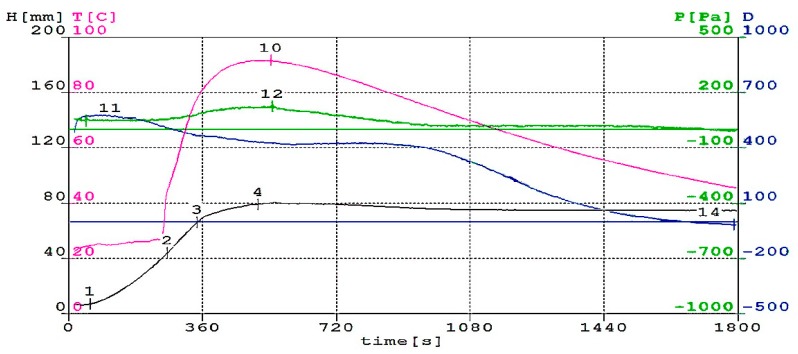
Change in height (H), temperature (T), pressure (p) and dielectric polarization (D) of reaction mixture during foaming process for M60 foam with 60 wt.% of biofiller.

The basic condition, which a well-designed mixture for synthesis of polyurethane/polyisocyanurate foam must meet, is a synchronization of the moment of maximum gas evolution with time. Then polymer reaches the appropriate viscosity. If the polyurethane has a too low viscosity, the foam will fall, because it will be impossible to keep the gas molecules in the polymer matrix. However, if the polymer matrix has a too high viscosity, the foaming process does not proceed correctly [37,38]. The analysis of foaming process based on the results of tests (such as: temperature, pressure, dielectric properties, foam rise height and processing times) is helpful in designing the optimal composition of the reaction mixture. Figure 2, Figure 3 and Figure 4 show the changes in height, temperature, pressure and dielectric polarization of reaction mixture during the foaming process for M0, M30 and M60 foams.

Addition of rapeseed cake to polyurethane material significantly influenced the course of the foaming process, by reducing the reactivity of the reaction mixture. One of parameters, which describes the reactivity of the system, is dielectric polarization. Its value provides information related to the course of gelation reactions and the rate of polyurethane matrix formation. Analyzing Figure 2, Figure 3 and Figure 4, it can be concluded that dielectric polarization (curve D) decreased as the reaction progressed for all considered reactions. The addition of oil cake to RPU/PIR foams contributed to a reduction of reactivity of systems and an elongation of reaction over time. Decreasing this parameter can be seen in curves of modified foams. Reduction of systems reactivity with an increasing amount of rapeseed cake in formulations confirmed the reduction of maximum foaming temperature. Reaching its maximum value took more time than in the case of reference foam. Maximum foaming temperature for reference foam was 132 °C. Use of biofiller in the amount of 30 wt.% contributed to decreasing of this parameter to 122 °C and to 86 °C for foam with 60 wt.% of rapeseed cake. A similar effect of reducing maximum temperature and dielectric polarization during the foaming reaction of foams modified with graphite and biopolyols was observed by Kurańska et al. [39] and Tu et al. [40]. In the case of the mentioned research, the foaming temperature of modified foams was from 110 °C to 160 °C. Changes in temperature were related to the fact that the amount of water in these systems remained the same for each system. Reaction of isocyanate groups with water is a highly exothermic reaction [41]. For systems that are only foamed with carbon dioxide produced in reaction between water and NCO groups, this largely determines the maximum temperature of the reaction mixture.

The pressure during foaming process was determined as a pressure force on the surface of the measuring table. Pressure value measured during foaming process decreased with increasing content of rapeseed cake in RPU/PIR foams. Pressure of foaming process is maximum 2608 Pa for reference foam (Figure 2), while for foams modified with a bio-filler it was 188 Pa and 122 Pa for M30 (Figure 3) and M60 (Figure 4), respectively. These changes can be related to the elongation of gelling reaction time, which caused large pressure changes. Such a system can expand in the long time, exerting pressure on the measuring table. Increasing biofiller content in foams also caused moving the value of maximum pressure in time. This value was reached by the unmodified formulation after 323 s. Addition of rapeseed cake extended this parameter to 330 s for a formulation with 60 wt.% of this filler. Obtained results were complied with the processing times of lignin-based polyurethane foams described by Gómez-Fernández et al. [42]. Foaming times proved that the use of particles of filler in PU foam mixtures increased the start times of the reaction and slowed the formation of cell structures.

Prepared systems containing different amounts of filler were characterized by the presence of maximum values of pressure, temperature, foam height and minimum value of dielectric polarization value at different times. It indicated a different course of foaming and gelation reactions. Foams in which rapeseed cake was used as a physical filler were characterized by the presence of maximum temperature at the time when the foam reached its maximum height. This effect is related to the fact that the reaction of isocyanate groups with water (during which the CO_2_ was generated) prevailed in the first step of reaction. In the next step, secondary chemical reactions took place with mixtures containing unreacted or excess isocyanate. Isocyanates react exothermically not only with compounds containing active hydrogen, but they can also react with each other under appropriate conditions. A special role in production of foams was also played by water. This component reacted with isocyanates and generated in-situ CO_2_, which was a blowing agent of obtained materials. The new chemical structures affected the properties of the obtained RPU/PIR foams. This process was hampered by use of large amounts of physical filler that hindered and slowed down the combining of formulation ingredients. Confirmation of this could be a different way of changes in dielectric polarization in comparison with foam without filler. Obtained results were confirmed by the analysis of processing times measured during the process of obtaining RPU/PIR foams. The cream, free rise, string gel and tack free times of modified foams all extended in comparison with those parameters for reference foam. Processing times of the obtained reference foam were 6 s for cream time, 15 s for free rise time, 28 s for string gel time and 31 s for tack free time. Elongation of these parameters in the case of modified foams was a consequence of addition of biofiller. The cream, free rise, string gel and tack free times for M60 foam were 17 s, 68 s, 80 s and 86 s, respectively. A similar dependence was observed by Kurańska et al. [43]. Authors stated that addition of vegetable fillers, e.g., wood fibers, reduces the reactivity of the polyurethane system, while contributes to elongation of processing times associated with obtaining PUR foams.

### 3.2. Cell Structure of RPU/PIR Foams and Thermal Insulation Properties

**Figure 5:**
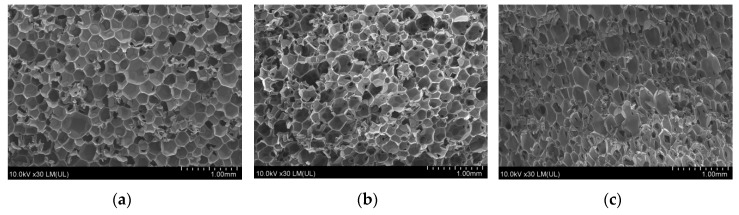
SEM micrographs of RPU/PIR foams surface (in parallel direction to foam rise) of reference foam (**a**) and foams modified by 30 wt.% (**b**) and 60 wt.% (**c**) of rapeseed cake.

One of the most important RPU/PIR foams parameters is their cellular structure. Morphological parameters, such as cell size, wall and ribs thickness, have a significant influence on the physical and mechanical properties of polyurethane foams [44,45]. The structure of RPU/PIR, in particular cell size and cell type (open or closed) depends mainly on the process parameters, such as viscosity of reaction mixture, pressure and temperature values during the foaming process [46]. According to Kang et el., appropriate synchronization of the reaction temperature with free rise and tack free times increases the key aspect of optimizing the foaming process. It can lead to the formation of foam with a well-developed cellular structure [47]. Cellular structure of the reference foam and foams modified by rapeseed cake in the amount of 30 and 60 wt.% was shown in Figure 5a–c.

The structure of reference foam M0 (Figure 5a) was regular with thin ribs. With increasing content of rapeseed cake in the formulation, the obtained foams were characterized by an increasingly heterogeneous structure with a higher content of open cells and thicker walls. Filler-modified foams had a reduced cell diameter in comparison with M0 foam. This parameter decreased from 316 μm to 270 and 260 μm for M0, M30 and M60 foams, respectively. This effect was related to the incorporation of the filler into the cell walls of polyurethane. Results of statistical analysis and comparison of SEM micrographs of M0, M30 and M60 foams, i.e., average cell size, average cell wall thickness and average content of closed cells per unit area, are shown in Table 3.

It is clearly visible in Figure 5a–c that the obtained foams had a closed cell structure with a slight tendency to open them in the case of foams with biofiller. Reference foam (M0) did not have a preferential orientation in the direction of cell growth. The cellular structure of unmodified foam was almost spherical and evenly distributed, with a small number of cells with broken walls. Micrographs of M30 and M60 foams are shown in Figure 5b,c. Modified foams showed a clear orientation of cells. This was particularly evident in the case of M60 foam, whose cells were elongated in the direction of foam rise. The reason for this was an increased viscosity of reaction mixture and the difficult expansion of cells after the addition of rapeseed cake. Filler particles can act as nucleation sites for cell formation. A larger number of cells begin to nucleate at the same time, therefore in foam is a larger number of cells with reduced diameter. A similar phenomenon was observed by Chang and Luo et al. [48,49]. Addition of powder filler can change the nucleation mode from homogeneous to inhomogeneous and reduce nucleation energy, which in turn promotes formation of a large number of small cells. The same dependence was also observed by Sung et al. [50].

Modification by rapeseed cake affected the obtaining of a less homogeneous structure with noticeable voids in foam structure. It was also clearly visible that the filler particles were evenly dispersed in the foam structure. Filler particles were built into the polyurethane matrix. It caused a thickening of cell walls. The consequence of this was the reduction of the cells size and an increase of wall thickness with increasing biofiller content (Table 3). In the case of RPU/PIR foams reinforced with rapeseed cake, phase separation was not observed for all compositions. Cell structure became less homogeneous and the cell walls became thicker with the addition of the filler.

One of the most important advantages of rigid polyurethane foams is their very good thermal insulation. This is related to the basic parameters used to assess this property—thermal conductivity coefficient (λ), content of closed cells, absorbability and water absorption of polyurethane matrix. The value of λ coefficient of PU foams should be below 0.035 W/(m·K). All obtained foams fulfilled this condition (Table 4).

Thermal insulation efficiency of rigid PU foams also depends on the content of closed cells. Based on the results presented in Table 4, it can be observed that the number of closed cells decreased from 87.9% for M0 foam to 79.1% for the foam with the highest rapeseed cake content (M60). Reduction in the number of closed cells have an impact on the increase in the thermal conductivity coefficient during long use of these foams, which in turn will result in poorer thermal insulation. This can be avoided by applying protective coatings on polyurethane elements, which prevents deterioration of thermal insulation properties. It is known that the share of thermal conductivity of gas contained in foam cells is about 60–80% of total value of the thermal conductivity coefficient of polyurethane foam. This depends primarily on the presence and type of blowing agent in cells and apparent density of foam [51]. The tendency to open cells and release blowing agent causes an increase in the thermal conductivity coefficient.

All of the obtained RPU/PIR foams were characterized by increased absorbability and water absorption, which is not a favorable feature in the case of thermal insulation materials. The water absorption of reference foam was 1.5%. Application of biofiller in formulation contributed to the increase of this parameter value to 10.9% for foam with the largest amount of rapeseed cake. A similar phenomenon was observed while testing absorbability of obtained foams. Water absorption increased from 10.9% for M0 foam to 56.6% for M60 foam. It is known that the intensity of water absorption by foam depends on polyurethane matrix and its cell structure [52]. Foam absorbed more water with increased content of biofiller. The reason for this was increase of open-cell structure, which was confirmed by SEM analysis. Therefore, it can be concluded that cellular morphology of RPU/PIR foams was the dominant factor affecting water sorption of analyzed materials. The second reason for the higher absorbability and water absorption capacity of obtained foams was also presence of rapeseed cake in cell walls, which was confirmed by SEM micrographs (Figure 5a–c) and conducted research (Table 4).

### 3.3. Physico-Mechanical Properties

Apparent density is one of the most important factors determining the mechanical properties of rigid PU/PIR foams. Thirumal et al. proved that increasing the apparent density of foam from 42 to 116 kg/m^3^, improved the compressive strength by about 800 kPa [53]. It was found based on conducted research that, together with increasing amount of rapeseed cake in the PU system, the apparent density of foams increased. It is worth noting that each of the formulations contained the same amount of water for production of a blowing agent. Table 5 presents apparent density, compressive strength and brittleness values for reference (unmodified) foam and foams modified by rapeseed cake.

**Figure 6:**
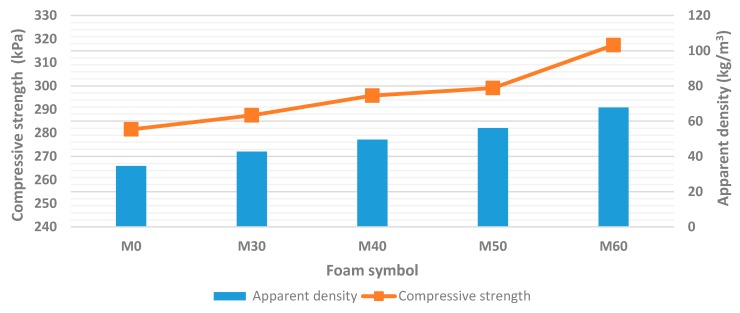
Dependence between content of rapeseed cake, apparent density and compressive strength in the parallel direction to foams rise.

Rigid PUR foams should have apparent densities above 30 kg/m^3^ in order to maintain adequate strength to withstand loads during various commercial applications (such as thermal insulation and reinforcement of structural elements) [54]. The apparent density of the unmodified foam was 34.6 kg/m^3^. Addition of ground rapeseed cake into the polyurethane composition increased apparent density of all modified foams. The highest increase in this parameter (up to 67.8 kg/m^3^) was observed in the case of foam containing 60 wt.% of bio-filler. Dependence between apparent density and compressive strength depending on the content of rapeseed cake was shown in Figure 6.

It can be concluded based on obtained test results that compressive strength of foams increased with increasing biofiller content. Reference foam had the lowest density of 34.6 kg/m^3^ and the lowest compression strength of 281.5 kPa. However, sample with the highest content of rapeseed cake (60 wt.%) had a density of 67.8 kg/m^3^ and compressive strength of 317.4 kPa. The reasons for this trend were primarily in the composition of the polyurethane formulation. During the research, polyurethane-polyisocyanurate foams were prepared using a chemical foaming method. Carbon dioxide was generated as a blowing agent during reaction of isocyanate group with water. The addition of rapeseed cake as a biofiller to obtaining foams increased the composition of solid phase with the same volume of generated carbon dioxide. It obviously caused an increase in density of obtained materials. In addition, the mechanical properties of foam were mainly related to its density. Thus, compressive strength increased with increasing apparent density of RPU/PIR foams [55,56]. 

In turn, the incorporation of biofiller and its homogeneous distribution in foam structure contributed to reinforcement of walls and ribs of the polyurethane matrix. It caused an increase in compressive strength. In addition, it was also necessary to take into account the chemical composition of filler. The high content of fiber caused a strengthening effect on the polymer matrix. A similar dependence was noted by Zhang et al. They used soy protein as a biofiller for RPU foams [57]. The small, closed cell structures that characterize foam with bio-filler also contributed to improvement of mechanical properties.

Addition of the oil cake to rigid polyurethane-polyisocyanurate foams resulted in an advantageous reduction of their brittleness. This parameter for reference foam M0 was 23.8%. The gradual addition of biofiller caused a gradual reduction in brittleness of modified foams. Reduction of this parameter to 10.2% was observed for foam with highest content of it. The lower brittleness of obtained polyurethane materials was related to reduction of cells size, strengthening of their walls with the biofiller and obtaining a compact structure, which was more resistant to mechanical damage. It can be concluded based on the results of strength tests that foams with the addition of rapeseed cake can be potentially used in the civil engineering and packaging industries.

### 3.4. Aging Resistance Tests

Aging resistance tests during use were carried out to predict resistance to changes in mass, dimensions and volume of the tested material during it use. This test was a simulation of aging during operation in a shortened time, usually from 24 to 72 h. The biggest changes occur in the above-mentioned parameters during this time [58]. Simulated aging of the obtained RPU/PIR foams was carried out in thermostating process of foams in 120 °C in a laboratory dryer with forced circulation for 48 h. Parameters like changes of linear dimension, changes of geometrical volume and mass loss were determined after thermostating the reference foam and foams modified by rapeseed cake. They were compared with the results before test and corresponding aging changes were obtained. The results of the percentage changes in linear dimensions, geometric volume and mass were presented in Table 6.

Changes in linear dimensions, geometric volume and mass loss are very important for using RPU/PIR foams in thermal insulation. Decrease of dimensional stability may result in the plasticization of polyurethane matrix by the blowing agents dissolving in it. The resistance of RPU/PIR foams to plasticization depends on the type of blowing agent used [59]. Using carbon dioxide as a blowing agent during the conducted research had significantly reduced the risk of susceptibility to plasticization of matrix and thus the occurrence of negative changes in the material during aging tests. It is assumed in industrial applications that polyurethane materials with closed cells due to aging should not change their linear dimensions by more than 1–1.5% and geometrical volume by more than 3% [60]. The changes during aging process of the polyurethane material can be of two types. The first type of change (marked with “−“ at the numerical value) is so-called shrinkage, i.e., decreasing the linear dimension or the geometrical volume of the foams. The second type of change (marked with the “+” at the numerical value) is increase of linear dimension or geometrical volume.

Changes in linear dimensions of foams modified by rapeseed cake (Table 6) were in the range of 0 to 0.9%. A change of −2.36% was noted for the unmodified M0 foam, which indicated a tendency to shrink. This was a very unfavorable phenomenon which significantly reduced the efficiency of insulation. In the case of the change of geometrical volume of the obtained materials, the advantageous effect of modifications on the structure stability was observed. This parameter for M0 foam was 2.75%, while its value for all foams containing rapeseed cake did not exceed 1%. RPU/PIR foam mass loss was a parameter related to material resistance to high temperatures. It indicated that physical (e.g., evaporation of water, diffusion of blowing agent) and chemical changes (e.g., degradation of material components) can occur. In the case of obtained foams, there was a correlation between mass loss and amount of rapeseed cake in formulation. The obtained compressive strength results confirmed that the increase in the share of rapeseed cake had strengthened the polyurethane matrix, which prevented diffusion of the blowing agent. Mass loss in reference foam M0 was 3.09%. The use of vegetable filler contributed to higher stability of this parameter at less than 1%.

### 3.5. Flammability and Thermal Resistance

One of the main disadvantages of rigid PU foams is their high flammability and low thermal resistance. Strict flammability requirements are imposed on foam materials for building applications. Obtaining low- or non-flammable materials requires multidirectional solutions both at the step of chemical structure design and use of flame-retardant additives [61,62,63,64,65,66,67]. PU foams based on petrochemical polyols are flammable and can be an additional source of fuel in case of a fire disaster. This is a major problem and limits the use of PU material [68,69]. Porous, light foams are flammable and have a tendency to quickly spread flame and high heat emission [70]. There are two basic processes during combustion of RPU/PIR foams: oxidation of gaseous fuel in the flame and oxidation of solid fuel on the surface of the foam. The flame is a result of the oxidation reaction of small-molecule compounds formed as a product of pyrolysis of the blowing agent and the PU matrix. Also important is heterogeneous oxidation of compounds in the surface layer of the burning material, which is significantly dependent on the oxygen concentration in atmosphere. Data in the literature indicate that over 90% of the heat causing degradation and destruction of the polymer provides oxidation reactions on its surface, while the flame alone provides only about 1% of heat [71]. It distinguishes three classes of combustibility of materials based on the value of limited oxygen index (LOI): flammable—LOI < 21%, fire retardant—LOI = 21–28% and non-flammable—LOI > 28% [15]. Rigid polyurethane-polyisocyanurate foams without the addition of a flame-retardant compound have a LOI value of up to 19–20%. Therefore, they are flammable according to the adopted classification. Flammability reduction of PU/PIR materials is usually achieved by adding different flame retardants, e.g., compounds containing boron, phosphorus, nitrogen atoms [71,72,73]. The lowest value of the oxygen index was noted for the M0 foam. It was equal to 21.4%. The value of this parameter increased to about 25% for all foams after addition of rapeseed cake. This value of limited oxygen index allowed including the obtained foams for fire-retardant materials.

**Figure 7:**
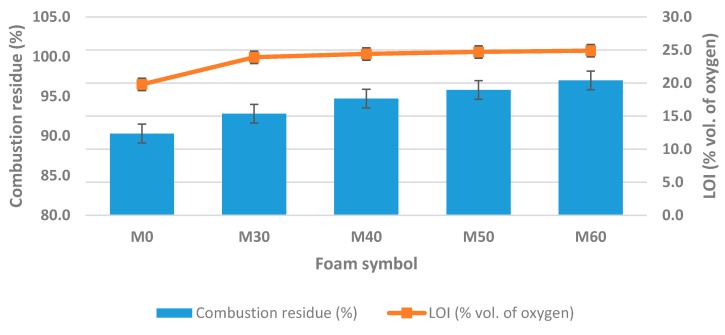
Dependence between content of rapeseed cake, combustion residue and limited oxygen index (LOI).

The formulation developed by authors made it possible to incorporate isocyanuric trimerization structures into PUR matrix by increasing the isocyanate index. Presence of a isocyanurate ring in polyurethane structure allowed the formation of a charred layer on the surface of the burning material. The charred layer was created at the expense of volatile combustible gases and acted as a protective barrier, which sealed the polymer matrix against the access of oxygen. It also prevented the release of volatile polymer pyrolysis products. Additionally, by adding rapeseed cake as a filler, nitrogen and phosphorus compounds have been added into the polyurethane structure (Table 1). These elements have been used as flame retardants for years. In the presence of phosphorus compounds, the chemical reactions associated with the combustion of RPU/PIR foam lead to the creation of crosslinked polyaromatic structures, which in turn are transformed into a carbonized product (coke). Addition of nitrogen compounds together with phosphorus compounds has a synergistic effect that increases flame retardant properties. Nitrogen strengthens the binding of phosphorus to the polymer matrix and simplifies the formation of a carbonized protective barrier [74]. Confirmation of flammability reduction effect in RPU/PIR foams, after addition of biofiller, was also obtained from results of a vertical flammability test. Dependence between combustion residue, limited oxygen index and amount of added rapeseed cake is shown in Figure 7.

Obtained results of combustion residues showed that a clear difference of this parameter was noted for RPU/PIR foams with and without rapeseed cake. Biofiller based materials were characterized by a much higher combustion residue than reference foam. Already 30 wt.% of this filler in the foam formulation significantly influenced the increase in this parameter (increase from 92.8% to 97.0% for modified foams, compared to 90.3% for reference foam). The reason for this phenomenon was the synergism of phosphorus and nitrogen which were a component of rapeseed cake. This meant that rapeseed cake acted as a flame retardant in a polyurethane formulation. Undoubtedly, also the combination of advantages of urethane and isocyanurate bonds in the foam composition had an impact on reduction of the flammability of polyurethane-polyisocyanurate foams. Isocyanurate bonds are more thermally stable than urethane bonds from the thermodynamic point of view (urethane dissociates at about 200 °C, as opposed to isocyanurate ring—350 °C) [75,76].

One of the main conditions that must be met by insulation materials used in civil engineering is good thermal resistance. Thermal resistance is associated with physical changes in PUR foam which occur under the influence of temperature. The increased temperature affects cracking of the weakest bonds in rigid polyurethane foams, causing degradation of the polymer. Resulting from this is a weight loss of the material. In order to characterize the influence of rapeseed cake on the thermal properties of foams, the obtained materials were subjected to thermogravimetric analysis (TGA) under nitrogen atmosphere at a temperature range of 20–1000 °C. TGA results of reference foam (M0) and foam with 60 wt.% of biofiller (M60) are shown in Table 7.

**Figure 8:**
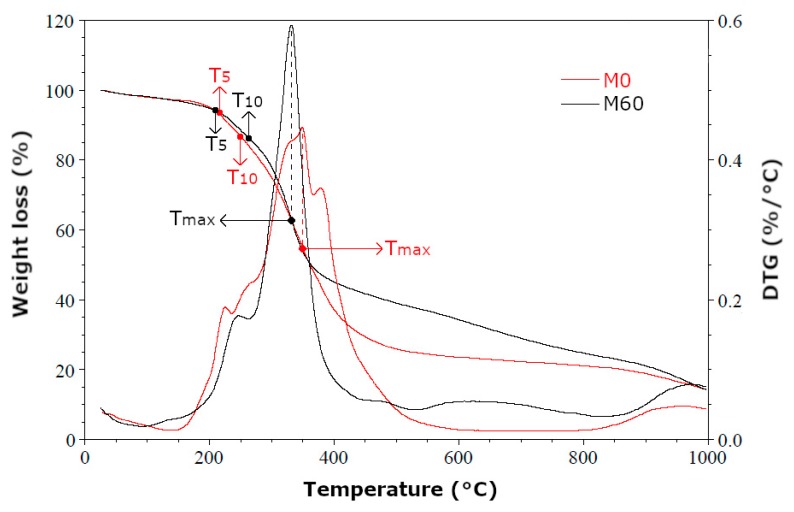
Thermogram of reference foam (M0) and modified foam containing 60 wt.% of bio-filler (M60).

Figure 8 showed the thermogram of M0 (reference foam) and M60 (foam with 60 wt % in relation to sum of masses of polyol and polyisocyanate raw materials) samples. Characteristic temperatures were marked: T_5_—temperature of 5% loss of weight, T_10_—temperature of 10% loss of weight, T_max_—temperature at which the highest loss of weight was occurred.

It was noticed on the thermograms of M0 and M60 foams that at a temperature of about 50 °C a weight loss of few percent occurred. It was associated with the migration of blowing agent (carbon dioxide) enclosed inside the polyurethane matrix into the environment. The course of TGA curves was the same to T_5_ (about 205 °C). A slight increase in thermal resistance of M60 foam was noted during the heating. The highest weight loss in the M0 foam occurred in temperature range from 200 to 610 °C. The maximum weight loss rate was 4.5 wt.%/min. In the case of M60 foam, the highest weight loss occurred in the range from 200 to 515 °C, with the highest weight loss rate of 5.9 wt.%/min. Many reactions occur in this temperature range, for example the dissociation of urethane bond and thermal decomposition of polyoxypropylene polyol (Rokopol RF-551) fragments, decomposition of carbodiimide, urea or ether bonds in used polyol. Also, isocyanurate rings are degraded down in this range. The narrower range of the highest weight loss and increased weight loss rate of the foam M60 was associated with thermal decomposition of organic substances contained in the rapeseed cake. Organic matter, like proteins, and fats contained in the biofiller were completely decomposed during heating. Products of foam thermal degradation were formed in solid, liquid and largely gas phase, as a result of heating of tested foams in an inert gas atmosphere. Ash residue (in 1000 °C) was obtained after heating in amount of 15 wt.% of the initial sample.

**Figure 9:**
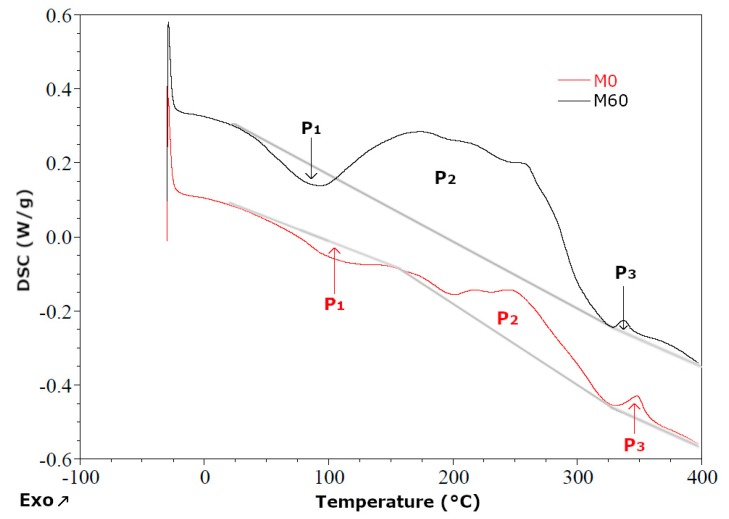
Differential scanning calorimetry (DSC) curves of reference foam (M0) and modified foam containing 60 wt.% of bio-filler (M60).

The course of DSC curves for samples M0 and M60 is shown in Figure 9. Differential scanning calorimetry was carried out in temperature range from −25 to 400 °C in a nitrogen atmosphere.

The M0 sample was RPU/PIR foam, which was used as a polymer matrix in foams modified with a bio-filler. Three peaks from the transformations occurring under influence of heating were observed on the DSC thermogram. The first of these was endothermic. It started at 40 °C and ran to 150 °C. It was related to the diffusion of blowing agent from inside of the foam. The other two peaks indicated that the transformations taking place from 150 to 320 °C and from 325 to 340 °C were exothermic. In these temperature ranges there were many transformations related to the degradation of chemical bonds present in the RPU/PIR foam, e.g., urethane bond hydrolysis, degradation of the ether bonds and polyoxypropylene polyol fragments, degradation of urea, carbodiimide and isocyanurate bonds. However, the energy effects of these changes are endothermic. Hydrolysis of urethane bonds results in compounds containing free hydroxyl (OH) and free isocyanate (NCO) groups. Low molecular weight substances containing OH groups are also released during the decomposition of polyether polyol. Degradation of isocyanurate rings leads to, among others, isocyanates. The presence of compounds containing OH and NCO groups leads to strongly exothermic reaction. Thus, the energy effect of the processes taking place was the sum of the endothermic effects of bonds decomposition reactions and exothermic effects resulting from the reaction of hydroxyl and isocyanate groups [33]. It was found on the basis of DSC curve of the M0 foam that the exothermic effect was dominant. The M60 sample was RPU/PIR foam with the highest content of rapeseed cake. Analogously to the M0, three peaks of transformation were observed on the DSC curve. The first and third transformation occurred at the same temperature ranges and did not show significant changes in the energy effect relative to reference foam. It meant that in both foams there were exactly two of the same transformations (including diffusion of blowing agent, etc.). The second transformation was more complex. Its total energy effect was influenced by both the reactions of the degradation of individual bonds in the foam, as well as reactions of OH groups with the obtained NCO groups and the degradation of organic substances contained in rapeseed cake. It was found on the basis of DSC curves that the exothermic effect was also dominant in this transformation. However, it was less intense than in the reference foam.

## 4. Conclusions

A formulation and method for obtaining rigid PU/PIR foams modified with waste from the oil industry (rapeseed cake) was developed as part of this research. RPU/PIR foams were prepared using the one-step method, from a two-component system in a chemical equivalent ratio of NCO:OH groups equal to 3.7:1. The use of a biofiller in the polyurethane system allowed to obtain PU materials with more advantageous and functional properties than commercially available foams. The increase in the content of rapeseed cake in polyurethane foams caused changes in the foaming process, e.g., reducing the reactivity of the system, decreasing its temperature and dielectric polarization and extending processing times. Modification of obtained polyurethane materials by rapeseed cake resulted in higher apparent density, compressive strength, absorbability and water absorption. Addition of a powder filler into the formulation made it possible to significantly reduce brittleness and increase resistance to aging. Biofiller used in the formulation influenced on the flammability of foams by increasing combustion residue and limited oxygen index. It was found that the developed method allowed fast, cheap and ecological management of rapeseed oil cake with the possibility of its re-use in a different form—as a factor reinforcing the matrix of rigid polyurethane-polyisocyanurate foams. Durable, low-brittleness, flame-retardant and environmentally friendly composite foams can provide promising advances in the design of new functional materials that can be used in various industries.

## 5. Patents

The research results are protected by the Polish Patent No. PL 227036.

## Figures and Tables

**Table 1:** The average chemical composition of rapeseed cake (per 1 kg of rapeseed cake).

Parameter	Dry Mass (%)	Proteins (%)	Minerals (%)	Vitamins (%)	Glucosinolates (%)	Fat (%)	Crude Fiber (%)
**Value**	93.13	38.00	11.99	0.70	0.12	15.48	12.60

**Table 2:** Formulation of rigid polyurethane-polyisocyanurate (RPU/PIR) foams with rapeseed cake.

Foam Symbol	Rokopol RF-551 (g)	Tegostab 8460 (g)	33% DABCO (g)	33% Potassium Acetate (g)	Antiblaze TCMP (g)	Distilled Water (g)	Rapeseed Cake (g)	Purocyn B (g)
**M0**	66.80	5.40	3.15	7.95	54.00	3.15	0.00	250.60
**M30**	66.80	5.40	3.15	7.95	54.00	3.15	95.20	250.60
**M40**	66.80	5.40	3.15	7.95	54.00	3.15	127.00	250.60
**M50**	66.80	5.40	3.15	7.95	54.00	3.15	158.70	250.60
**M60**	66.80	5.40	3.15	7.95	54.00	3.15	190.40	250.60

**Table 3:** Results of SEM micrographs analysis.

Foam Symbol	Cell Size (μm)	Thickness of Cell Wall (μm)	Content of Closed Cells per Area Unit (cells/mm^2^)
M0	316 ± 15	17 ± 2	12 ± 1
M30	270 ± 14	20 ± 2	11 ± 1
M60	260 ± 12	24 ± 2	10 ± 1

**Table 4:** Thermal insulation properties of RPU/PIR foams modified by rapeseed cake.

Foam Symbol	λ (mW/(m·K))	Closed Cells Content (%)	Absorbability (%)	Water Absorption (%)
**M0**	34.1 ± 0.1	87.9 ± 0.9	10.9 ± 0.2	1.5 ± 0.1
**M30**	34.7 ± 0.1	82.6 ± 1.1	25.6 ± 0.6	4.2 ± 0.2
**M40**	34.6 ± 0.1	80.8 ± 0.8	31.3 ± 0.5	4.5 ± 0.2
**M50**	34.9 ± 0.1	79.7 ± 1.3	45.6 ± 0.9	5.8 ± 0.2
**M60**	34.8 ± 0.2	79.1 ± 1.2	56.6 ± 1.2	10.9 ± 0.3

**Table 5:** Values of apparent density, compressive strength and brittleness of obtained RPU/PIR foams.

Foam Symbol	Apparent Density (kg/m^3^)	Compressive Strength (kPa)	Brittleness (%)
**M0**	34.6 ± 0.4	281.5 ± 3.9	23.8 ± 0.9
**M30**	42.8 ± 0.7	287.5 ± 5.1	22.0 ± 0.6
**M40**	49.5 ± 0.8	295.9 ± 4.8	20.3 ± 0.6
**M50**	56.1 ± 1.1	299.1 ± 6.3	15.5 ± 0.4
**M60**	67.8 ± 1.2	317.4 ± 7.2	10.2 ± 0.3

**Table 6:** Results of linear dimension changes, geometrical volume changes and mass loss of obtained foams.

Test	M0	M30	M40	M50	M60
**Δ*l*** **(%)**	−2.4 ± 0.1	+0.9 ± 0.1	+0.9 ± 0.1	+0.7± 0.1	+0.8 ± 0.1
**Δ*V*** **(%)**	+2.8 ± 0.1	+1.0 ± 0.1	+0.5 ± 0.1	+1.0± 0.1	+1.0 ± 0.1
**Δ*m*** **(%)**	3.1 ± 0.1	1.0 ± 0.1	0.2 ± 0.0	0.5 ± 0.0	0.1 ± 0.0

**Table 7:** Results of thermogravimetric analysis (TGA) of reference foam M0 and M60 foam—containing 60 wt.% of rapeseed cake.

Foam Symbol	T_5_ (°C)	T_10_ (°C)	T_max_ (°C)	Highest Weight Loss (%/°C)	Highest Weight Loss Rate (%/min)	Residue (%)
**M0**	206	249	359	0.45	4.5	15
**M60**	204	264	347	0.59	5.9	15
